# Effect of School-Based Home-Collaborative Lifestyle Education on Reducing Subjective Psychosomatic Symptoms in Adolescents: A Cluster Randomised Controlled Trial

**DOI:** 10.1371/journal.pone.0165285

**Published:** 2016-10-25

**Authors:** Junko Watanabe, Mariko Watanabe, Kazue Yamaoka, Misa Adachi, Asuka Nemoto, Toshiro Tango

**Affiliations:** 1 Minami Kyushu University, Miyazaki, Japan; 2 Prefectural University of Kumamoto, Kumamoto, Japan; 3 Showa Women's University, Tokyo, Japan; 4 Teikyo University Graduate School of Public Health, Tokyo, Japan; 5 Nutrition Support Network LLC, Kanagawa, Japan; 6 Center for Medical Statistics, Tokyo, Japan; TNO, NETHERLANDS

## Abstract

In this study, we aimed to assess the effectiveness of a school-based home-collaborative lifestyle education program for adolescents (PADOK) in reducing poor subjective psychosomatic symptoms (SPS). The study was designed as a two-armed parallel cluster randomised controlled trial and the study population comprised adolescent students (aged 12–14 years, *n* = 1,565) who were recruited from 19 middle schools in Japan. The PADOK intervention or usual school programme was provided in schools to all eligible participants. The primary outcome was the SPS score at 6 months, while secondary outcomes included lifestyle factors, BMI, and dietary intakes. Analyses were undertaken on an intention to treat (ITT) basis accounting for the clustered design. Nineteen schools were randomised to the PADOK group (10 schools) and control group (9 schools). The numbers of students used for analysis were 1,509 for ITT and 1,420 (94.1%) for PPS. At 6 months, the crude mean change from baseline of the SPS scores by ITT analysis showed a significantly greater reduction in the PADOK group compared to that in the control group (−0.95, 95% *CI* −1.70 to −0.20, *P* = 0.016), while those for baseline-adjusted and multivariate-adjusted values showed similar directionality but were not significant (*P* = 0.063 and *P* = 0.130). The results indicated that the PADOK program may improve poor SPS scores among adolescents.

## Introduction

Adolescence is a critical stage of the life course that offers the opportunity to improve or prevent potential chronic health problems such as obesity [1–3], metabolic syndrome [4], and adverse psychological (psychosomatic or psychiatric) symptoms [5,6], both in adolescence and later in life [7]. Many school-based cluster randomisation trials have been conducted in Western societies to prevent obesity regarding lifestyle behaviour modifications among adolescents, but few studies have been conducted in Asia [3]. Improving the well-being of adolescents is a major public health concern [8] and requires effective lifestyle and behavioural interventions that target healthy dietary intakes [9,10] and physical activity among adolescents [11–14].

Not only obesity but also mental health problems have been reported to affect 10–20% of children and adolescents worldwide [15]. Although associations between adolescent lifestyles and poor subjective psychosomatic symptoms (SPS) scores have been reported [16,17], there have been few studies examining the effectiveness of interventions to alter adolescents’ lifestyle on improving their SPS scores [18,19]. In particular, very few relevant cluster randomised controlled trials (RCTs) have been conducted in Japan. Considering the rapid increase of poor SPS scores in Japan [20], it is important to develop an effective lifestyle and behaviour intervention program for general use with adolescents in Japan.

A lifestyle intervention program could be targeted to impact a broad range of outcomes related to improving the poor SPS scores of Japanese adolescents, including an increased enjoyment of school life and improving balanced dietary intakes and daily habits such as maintaining a regular sleeping routine. Considering these points, we developed a school-based home-collaborative lifestyle education program to reduce poor SPS in adolescents (**P**rogram for **ADO**lescent of lifestyle education in **K**umamoto, PADOK). The design of PADOK was based on strategies described in previous studies [9, 21–25] and aimed to change adolescents’ lifestyle in a non-compulsory way and provide feedback on their dietary habits by assessment using the FFQW82 food frequency questionnaire, which was developed to assess habitual dietary intake by evaluating the nutritional intake at each meal [22,23]. The inclusion of home-collaborative support may help to obtain favourable effects. We hypothesised that adolescents who participated in the PADOK group would show a greater improvement in their SPS scores than those who participated in the control group after 6 months. Correspondingly, the aim of this study was to assess the effectiveness of the PADOK intervention programme in reducing the frequency of poor SPS scores among adolescent students at 6 months after baseline compared with the usual education provided to Japanese middle school students by their schools.

## Methods

### Study design

**Fig 1:**
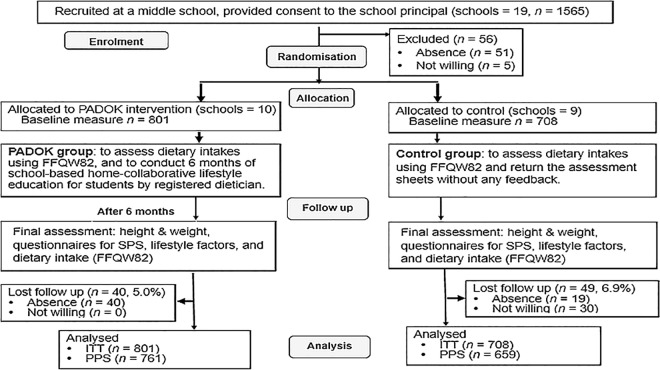
Consort flow diagram.

The study was designed as a two-armed school-based parallel cluster RCT, with individual middle schools as the unit of allocation and individual participants as the unit of analysis. This study was conducted according to the guidelines laid down in the Declaration of Helsinki and all procedures involving human subjects/patients were approved by the Medical Ethics Committee of the Prefectural University of Kumamoto in 2012 (No. 24001) and was registered with the Japanese Clinical Trial Registry (UMIN 000012525). The registration had been late for the enrolment of participants (the participant recruitment period: May 2013 to July 2013) because it took time for the coordination with school teachers. The registration had been completed during the follow-up period (June 2013 to January 2014). Written informed consent was obtained from all subjects (middle school students) and their parents or guardians. The study followed the CONSORT guidelines for the design and reporting of clinical trials (S1 CONSORT Checklist), and the CONSORT flow diagram for the study is shown in Fig 1.

### Participants

Nineteen schools among 178 schools with a middle school in Kumamoto prefecture, Japan (around 52,000 students), were eligible for inclusion and were invited to participate. All adolescents in middle school years 1 and 2 (aged 12–14 years) in the participating schools were eligible unless they were not attending school (for example, absence due to sickness) or were not willing to participate.

Once schools had agreed to participate in the study, we sent a letter and information sheet about the study to the parents/guardians of the adolescent students at the school with an option to decline consent for their child to undertake the measurements (height and weight, dietary intakes [FFWQ82], lifestyle, and SPS assessment [self-reported questionnaire]) at baseline and at the end of the 6-month study period. Written informed consent was obtained from all subjects (middle school students: *n* = 1,565) and their parents or guardians. The adolescents whose parents/guardians did not decline consent were recruited and provided consent to their school principals.

### Randomisation, allocation concealment, and blinding

Once the schools had been recruited we randomly allocated them into the PADOK group and usual school programme (control) group using a permuted-block technique with the use of a randomisation list (random permutated blocks with block size 2).

Due to the nature of the treatment, it was not possible to blind subjects to the lifestyle education. However, team members of data management, with the exception of the project coordinator and research assistants, remained blind to the group allocations until the code was open after the last follow-up call was completed and the data were recorded. The project coordinator and research assistants were not blinded because they were responsible for distributing the education to participants.

To minimise the risk of bias, strict protocols for follow-up assessment procedures (in Japanese) were developed and research assistants were trained to adhere to these protocols.

### Interventions

Interventions were delivered in the academic year May 2013–January 2014 during health education classes.

#### PADOK program

**Fig 2:**
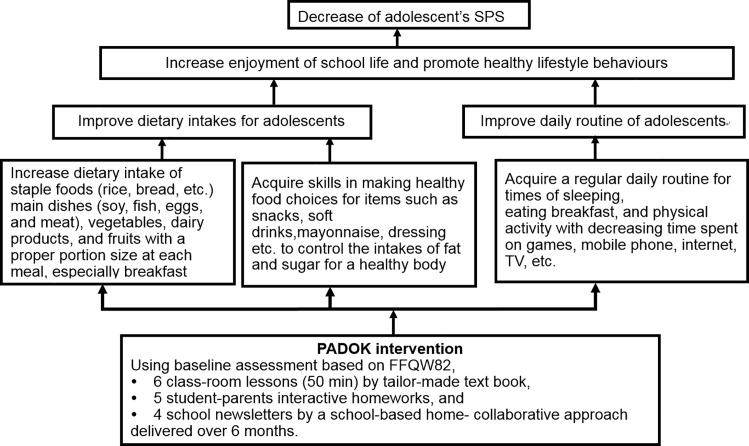
PADPK intervention scheme.

The PADOK intervention scheme is shown in Fig 2. Based on the assessment of dietary intakes using FFQW82, the PADOK program was administered with the aim of reducing poor SPS among the adolescent students. The PADOK intervention consisted of the programme of 6 classroom lessons, 5 student-parents interactive homework plans, a tailor-made text book for the lessons and homework assignments, and 4 school newsletters over a 6-month period.

As teaching materials, a tailor-made text book “Power up Dietary Lifestyle” and PowerPoint presentations were delivered to students, their school teachers, and registered dietitians, as well as learning support assistants. The details were summarised elsewhere [26]. The PADOK program also aims to improve students’ behaviour and lifestyle habits. Namely, increasing their enjoyment of school life and health, forming a habit of sleeping at regular hours of each day for a period of more than 6 hours, eating breakfast, and increasing their physical activity by decreasing the time spent on computer games, mobile phones, internet, and watching television. The program also encouraged students to increase their dietary intake of staple foods (rice, bread, etc.), main dishes (soy, fish, eggs, and meat), and vegetables, particularly at breakfast. The program also aimed to develop students’ skills in food selection by delivering advice and education, for example, not to consume high-fat foods such as mayonnaise and dressings or high-energy drinks.

#### Usual care

Students in the usual school programme (control) arm participated in health education sessions provided by the school according to the usual curriculum. The sessions were provided by the teacher with an external appointment for dietary assessment using the FFQW82. The usual care comprised any existing health curriculum about diet and/or exercise that is routinely taught at each participating school. An overview of the study is shown in Fig 1.

### Training for registered dietitians and learning support assistants

Training for registered dietitians and learning support assistants was provided by the trial leader, who is a registered dietitian. The training took place over a whole day (8–10 hours) at the study managing centre. During the training days, the rationale for the intervention was explained, and each lesson and homework activity was discussed interactively.

Four trained facilitators led each session working alongside the registered dietitian. The facilitators had at least an undergraduate university degree in a relevant discipline, an appropriate professional background, or experience of working with adolescents. In all sessions, the intervention was delivered under observation by the facilitators.

### Outcome measures

Outcomes were collected during class time by questionnaires administered by researchers and completed by students at baseline and 6 months. The primary outcome was the SPS score measured by the responses to the questionnaire. The SPS questionnaire consists of 9 symptoms (fatigue, headache, lassitude, irritation, lack of concentration, lack of motivation, poor ability to wake in the morning, upset stomach, and stiff shoulders). The Japanese and English translated versions of the questionnaire are shown in S1 Fig. The response categories for the frequency of experiencing each symptom were “0 = never”, “1 = rarely”, “2 = sometimes”, “3 = often”, and “4 = always.” Assuming the Likert scale, we calculated the SPS score (0–36 points) as a sum of the category values for 9 items. Because the assumption of the Likert scale was not necessarily guaranteed, we calculated another score (SPS-D score) as a sensitivity analysis. Since the response “never” should be desirable for adolescents, a response in any of the other 4 categories was classified as “having poor symptoms”. The number of poor symptoms for which students were classified as “having poor symptoms” was used as the SPS-D score (0–9 points). For both the SPS and SPS-D scores, a higher score denotes poorer symptoms. Internal consistency of the SPS score was examined by the Cronbach’s α coefficient. The results for the validity and reliability of the SPS score are shown in S2 Fig.

Secondary outcomes were lifestyle factors including enjoyment of school life, BMI, and dietary intakes assessed by the FFQW82 (*see* Table 1). The FFQW82 consists of a list of 82 foods with colour illustrations showing portion sizes, and the dietary habits of respondents for the previous 1 month can be calculated for each meal (breakfast, lunch, and dinner) and by food group. The validity and reliability of FFQW82 were previously established for adolescents [23]. The Japanese and English translated versions of the FFQW82 are shown in S3 Fig. The details for the FFQW82 and the assessment method were reported elsewhere [26] and the Japanese version of the dietary assessment software using FFQW82 can be downloaded from the publisher’s website [26].

**Table 1:** Baseline characteristics of participants allocated to intervention group or control group (n = 1,509).

	Intervention		Control		
	10 schools (n = 801)		9 schools (n = 708)(n = 708)		P-value
Sex (% boys)	382	47.7%	335	47.3%	0.812
BMI (means, SD)	19.2	2.9	19.0	2.9	0.333
SPS score (means, SD)	23.2	3.9	22.8	6.6	0.248
Lifestyle factors (n, % of "always")					
Enjoying school life (very much)	54	6.8%	50	7.1%	0.816
Taking exercise and stretching	442	55.2%	430	60.7%	0.029
Fast asleep at 12 AM (midnight)	401	50.1%	369	52.1%	0.425
More than 6 hours sleep	452	56.4%	417	58.9%	0.333
Health condition (very good)	275	34.3%	266	37.6%	0.191
Staple food consumed per breakfast	686	85.6%	588	83.1%	0.166
Main dishes consumed per breakfast	257	32.1%	210	29.7%	0.309
Vegetables consumed per breakfast	156	19.5%	176	24.9%	0.012
Main dishes consumed per lunch	326	40.9%	292	41.3%	0.860
Vegetables consumed per lunch	321	40.1%	319	45.1%	0.053
Dairy products consumed per day	385	48.1%	372	52.6%	0.082
Not consumed fatty foods	247	30.8%	242	34.2%	0.160
Not consumed snacks after 10 PM	472	59.0%	403	56.9%	0.414
Dietary (energy) intakes (mean, SD)					
Whole day (kJ)	7224	1414	7216	1569	0.918^$^
Breakfast (kJ)	1635	360	1646	397	0.590^$^
Lunch (kJ)	2511	858	2514	941	0.950^$^
Dinner (kJ)	3t117	456	3103	452	0.540^$^

SPS, subjective psychosomatic symptoms; SD, standard deviation; P-values were calculated using *t*-test for continuous variables and chi-square test for categorical variables except for $ (Wilcoxon rank sum test).

### Study hypothesis

The hypothesis underlying this study was that adolescents who participated in the PADOK group would show a greater decrease in their mean SPS score than adolescents who participated in the control group after 6 months. The effects of the intervention were expected to be measured after 6 months using a self-completed questionnaire.

### Sample size

The sample size required for the study was determined based on the information needed to detect a difference in the primary outcome with a significance level of 5% and power of 80%, under the assumptions of 40 students per cluster (assuming the same sample size for each cluster), an effect size (for poor SPS) of 0.3 (Cohen’s d), and an intraclass correlation coefficient of 0.02. The effect size was estimated from our experience in a former study [27].

### Data management and data monitoring

We captured all study data in a Microsoft Excel file. The validation rules for each case record had been pre-specified and included range checks so that inaccuracies in data collection could be identified at an early stage.

### Statistical analysis

We used descriptive statistics to assess the balance between the trial arms at baseline. To ensure that cluster randomisation was successful, the significance of differences between the intervention and control groups was examined using chi-squared test and *t*-test. The primary effect was assessed by calculating the difference between the changes from baseline to 6 months in the SPS scores of the PADOK and control groups. The primary analysis was conducted based on an intention to treat (ITT) principle with the full analysis set and imputation of missing data was performed using the last observation carried forward method (ITT/LOCF) and a multiple imputation method (ITT/MI) using chained equations under the assumption of missing at random [28]. A linear random-effects mixed model employing the maximum likelihood method was used for the analyses. A general linear random-effects mixed model employing the restricted maximum likelihood method was used for the analysis of continuous variables. Outcome measures were used to examine the effects of the intervention by a crude model (Model 1), a model adjusted for baseline values (Model 2), and a multivariate-adjusted model (adjusted for baseline, sex, age, and BMI) (Model 3).

Secondary analyses were conducted for the secondary outcomes using the ITT/LOCF approach. Sensitivity analyses were conducted for the primary and the secondary outcomes using the per protocol set (PPS) identified from the complete data set following the criteria determined *a priori* including analyses for the SPS-D score for ITT/LOCF. As for the dichotomous secondary outcomes, a generalised linear random-effects mixed model (logistic model) was used for the analysis and associations were shown as an odds ratio and its 95% confidence interval (CI).

All tests for significance were conducted using a 2-sided approach with a 5% significance level. All statistical analyses were performed using SAS version 9.4 for Windows (SAS Institute, Cary, NC, USA).

## Results

### Baseline characteristics

We initially approached 178 schools to request their participation in the study and among them, 19 schools agreed to participate, while 159 schools declined. The most common reason given was that “the curriculum has already been fixed and cannot be changed”. Fig 1 shows the trial flow chart. Of the 1,565 potentially eligible students in the 19 participating schools who provided consent to their school principal, 56 students (absence = 51, not willing = 5) were excluded before randomisation and did not undergo baseline data collection.

Students who had provided written informed consent and completed baseline assessments were enrolled into the study. The 19 participating schools were randomised to the PADOK group (10 schools) and the control group (9 schools). The number of students enrolled was 1,509. After 6 months, 1,420 participants completed the final assessments of height, weight, SPS, lifestyle factors, and dietary intakes (FFQW82). Of the remaining 89 participants, 59 were absent (PADOK group *n* = 40, control group *n* = 19) and 30, all in the control group, were not willing to attend their 6-month follow-up appointment.

Table 1 shows the baseline characteristics of the participants assigned to the PADOK and control groups. The proportions of boys were 47.7% for the PADOK group and 47.3% for the control group. The mean (standard deviation, SD) BMI values were 19.2 (2.9) for the PADOK group and 19.0 (2.9) for the control group.

The Cronbach’s α coefficient of the SPS score was 0.88. The SPS scores at baseline were 23.2 (3.9) for the PADOK group and 22.8 (6.6) for the control group. The proportions of participants having each measured lifestyle factor and the energy intakes (kJ) were not largely different between the groups at baseline. There was a statistically significant difference (*P* = 0.012) between the intervention and control groups at baseline for ‘vegetables consumed per breakfast’. The control group reported a higher frequency for this habit.

### Intervention effects on the primary outcome

The mean change from baseline in the SPS score at 6 months as assessed by ITT/LOCF analysis was significantly reduced in the PADOK group compared with the control group for the crude mean difference (−0.95, 95% CI −1.70 to −0.20, *P* = 0.016). Reductions (i.e., negative changes) in the SPS score indicate improvements in the SPS of the intervention group compared with that of the control group. The mean changes from baseline for the baseline-adjusted (−0.72, 95% CI −1.48 to 0.04, *P* = 0.063) and multivariate-adjusted (−0.68, 95% CI −1.58 to 0.22, *P* = 0.130) values showed similar directionality but were not significant (Table 2). The results obtained by the ITT/MI method were similar to these (S1 Table). In the case of the SPS-D score, the mean changes from baseline were significant for the crude, baseline-adjusted, and multivariate-adjusted values (S2 Table). In addition, the sensitivity analyses yielded similar results for each analysis (S3 Table).

**Table 2:** Mean change of the SPS score from baseline at 6 months (intervention effect on primary outcome).

Total	ITT/LOCF (n = 1,509)				
	Difference	SE	95% CI		P-value
**Model 1**	−0.95	0.36	−1.70	−0.20	0.016
**Model 2**	−0.72	0.36	−1.48	0.04	0.063
**Model 3**	−0.68	0.43	−1.58	0.22	0.130

SPS, subjective psychosomatic symptoms; ITT/LOCF, Analysis by intention-to-treat principles performing imputation of missing data using the last observation carried forward method; SE: standard error; Model 1, crude mixed model; Model 2, mixed model adjusted for baseline; Model 3, mixed model adjusted for baseline, sex, age, and BMI.

### Intervention effects on the secondary outcomes for lifestyle habits

In the PADOK group, some of the measured lifestyle habits were improved according to the results of the ITT/LOCF analyses. These improvements (odds ratio [OR] < 1 for Model 2 and Model 3) were in the categories “Enjoying school life” (OR [95% CI]: 0.55 [0.33 to 0.92], *P* = 0.022 and 0.52 [0.33 to 0.84], *P* = 0.008), “Staple food consumed per breakfast” (0.69 [0.50 to 0.96], *P* = 0.028 and 0.68 [0.48 to 0.65]), “Main dishes consumed per breakfast” (0.69 [0.50–0.96], *P* = 0.025), and “Vegetables consumed per breakfast” (0.65 [0.45–0.93], *P* = 0.018). Those for Model 3 were similar to this (Table 3).

**Table 3:** Effects of lifestyle change at 6 months on resolving lifestyle factors (ITT/LOCF, n = 1509, intervention effects on secondary outcomes; baseline-adjusted odds ratio [Model 2] and multivariate-adjusted odds ratio [Model 3]).

Lifestyle factors	ITT/LOCF (n = 1,509)							
	Model 2				Model 3			
	OR	95% CI		P-value	OR	95% CI		P-value
		Lower	Upper			Lower	Upper	
**Enjoying school life (very much)**	0.55	0.33	0.92	0.022	0.52	0.33	0.84	0.008
**Taking exercise and stretching**	1.04	0.77	1.42	0.792	1.03	0.74	1.44	0.853
**Fast asleep at 12 AM (midnight)**	0.99	0.67	1.47	0.971	0.98	0.72	1.33	0.893
**More than 6 hours sleep**	0.87	0.69	1.10	0.257	0.85	0.67	1.09	0.198
**Health condition (very good)**	0.88	0.65	1.19	0.389	0.86	0.62	1.18	0.351
**Staple food**^**1)**^ **consumed per breakfast**	0.69	0.50	0.96	0.028	0.68	0.48	0.95	0.025
**Main dishes**^**2)**^ **consumed per breakfast**	0.69	0.50	0.96	0.025	0.69	0.51	0.93	0.014
**Vegetables consumed per breakfast**	0.65	0.45	0.93	0.018	0.64	0.47	0.88	0.005
**Main dishes consumed per lunch**	0.91	0.56	1.47	0.702	0.93	0.58	1.50	0.766
**Vegetables consumed per lunch**	1.10	0.80	1.52	0.565	1.11	0.80	1.53	0.528
**Dairy products consumed per day**	0.77	0.53	1.14	0.191	0.79	0.57	1.09	0.151
**Not consumed fatty foods**	0.89	0.61	1.29	0.524	0.88	0.61	1.26	0.477
**Not consumed snacks after 10 PM**	1.09	0.81	1.48	0.568	1.07	0.79	1.45	0.656

OR (odds ratio) < 1 indicates favourable for resolving lifestyle factors; 1) Staple food: rice, bread, etc.; 2) Main dishes: fish, soy, eggs, meat, etc.

The results obtained by the sensitivity analyses (PPS analyses) showed mostly similar results to the above (see S3 and S4 Tables). There was no significant difference in dietary intakes assessed by FFWQ82 between the PADOK and control groups (data not shown).

## Discussion

Our study assessed the effectiveness of a middle school-based home-collaborative lifestyle education program (PADOK) in reducing the frequency of poor SPS among Japanese middle school adolescents. The findings suggested that the PADOK intervention program was effective for improving the SPS score of adolescents. Improvements were also observed in some lifestyle habits, including increases in subjective enjoyment of school life, the daily servings of staple foods, main dishes, and vegetables consumed at breakfast.

This study provides the first evidence for improvements in SPS scores and lifestyle habits as a result of delivering a lifestyle education program, based on a cluster RCT in Japan. In particular, it is important to ascertain the breakfast habits of the adolescent age group [29]. The PADOK intervention is mainly focused on the benefits of regularly eating a healthy breakfast for reducing students’ SPS scores. Other reports on the beneficial effects of similar interventions such as the development of psychosocial and behavioural function [30], improved academic performance [31], decreases in tardiness and suspensions, and improved student behaviour and attentiveness may support our results. The potential mechanisms that could be targeted to improve adolescents’ SPS scores include fostering an increased enjoyment of school life, a balanced dietary intake, and healthy daily habits.

One recent cross-sectional study conducted in England provided information about changing mental health trends in early adolescence and specifically identified a significant increase in emotional problems in girls aged 11–13 years [32]. Our findings were concordant with those of previous studies, which reported that students who regularly ate breakfast were more likely to behave better in school and get along with their peers better than those who did not [33]. Many therapeutic lifestyle changes, such as meditation, relaxation, recreation, and time in nature, are enjoyable and may therefore become self-sustaining healthy habits [34].

Our results showed that the PADOK intervention led to a reduction in the mean SPS score of students. In the school-based home-collaborative lifestyle education, registered dieticians encouraged students and their parents or guardians to help students to improve their lifestyle through each classroom lesson, homework assignment, and newsletter by following a cycle of goal setting, practice, and assessment. Our study focused on treating poor SPS as a whole entity, while previous studies [18, 19, 35] involving RCTs focused on treating certain individual mental health outcomes such as having depression. These studies either administered a questionnaire for self-completion by the participants [18, 19] or assessed risk factors and protective factors related to having depressive symptoms [35]. In these studies, no significant differences between the intervention and control groups were observed. A prior systematic review reported that there was limited evidence that the school environment has a major influence on the mental health of adolescents, although student perceptions of teacher support and school connectedness were associated with better emotional health [36]. On the other hand, Williams and her colleagues [37] conducted a systematic assessment of the school environment and identified the opportunity to design realistic and relevant interventions for promoting health in adolescents through improving physical activity and nutrition behaviours using the PRECEDE framework. We cannot exclude the possibility that the teachers of students in the PADOK group had changed their way of thinking about the importance of lifestyle education and aimed towards the improvement of such education in their general classes. If so, we can consider it as a secondary effect of the PADOK intervention. Further study is needed to elucidate the detailed mechanisms by which PADOK is effective in reducing SPS and promoting health-promoting lifestyle behaviours.

### Strengths and limitations of this study

We considered it important to involve students’ parents or guardians in the intervention [38]. To achieve this, the students were asked to discuss the knowledge gained from the PADOK program with their parents/guardians. The inclusion of home-collaborative support may help to obtain favourable effects. In a previous clustered RCT for physical activity intervention among adolescents, an intervention that included parental support led to an increase in self-reported school-related physical activity [12, 39]. In our study, the textbook was used for communication among the student, his or her guardian(s), and the registered dietitian. Free exchange of information among the 3 persons concerned was possible by using the notes in the text book. Thus, it was hoped that the process might create a shared understanding among these 3 persons.

Our study had several limitations. Firstly, the success of the PADOK program was to some extent dependent on the skill of the dietitian. To address this issue, we developed a training process for registered dietitians to undertake before the start of the randomised study. Secondly, we relied on self-reported ratings of SPS and did not undertake any diagnostic interviews. It is therefore possible that there were important changes in the status of SPS. The results of our ITT/LOCF and ITT/MI analyses denoted significance only for crude differences from baseline. The directionality of the differences was similar in the case of the SPS score assuming the Likert scale, but these differences did not reach significance. Since we used the LOCF method for missing values, the statistical analysis might be biased towards being conservative. However, such a conservative bias is generally preferable to lower the possibility of obtaining false positive results in a clinical trial. Further, we added the SPS-D score that was calculated as the number of responses for “having poor symptoms”, since the response “never having poor symptoms” should be desirable for adolescents. The construct validity was established (see the results of factor analyses [EFA and CFA] in S2 Fig) for both scoring methods. When we calculated the SPS and SPS-D scores, Cronbach’s α coefficients were 0.88 and 0.90, respectively. From these results, the scoring methods we used in this study were proved to be valid. In addition, we conducted multiple testing for the secondary outcomes, and we should, therefore, interpret the significance of the results carefully.

Thirdly, the generalisability of the results was limited to Japanese middle school students in Kumamoto prefecture in Japan. In addition, we initially approached 178 schools to request their participation in the study and among them, 19 schools agreed to participate, while the remaining 159 schools declined. The most common reason given was that “the curriculum has already been fixed and cannot be changed”. Therefore, the sample set included in this trial may over-represent schools that are more innovative than average and this may be a risk of bias. However, we conducted randomisation and the risk of bias is small, although we cannot deny that a risk exists. Considering that the health and activity conditions among the students in Kumamoto were not largely different from those of average Japanese adolescents according to the available data [20], the results may also apply to general adolescents in other prefectures in Japan. Fourthly, the term of intervention was 6 months in this study, although longer term interventions have been found to improve the mental health of pupils [40]. As for the duration of the intervention, there are many types of study design [2]. Considering the feasibility of the real education situation, we considered it as practicable. In addition, there was also emerging evidence that health behaviour interventions might have "spill-over effects" on mental health outcomes [41, 42], which we should also consider. Further studies are needed to assess the long-term effects and cost-effectiveness of the PADOK program. Fifthly, to ensure that cluster randomisation was successful, we examined the differences in baseline values between the intervention and control groups. Although some of the variables showed significant differences, we think these differences may have been caused by chance because of the nature of randomisation. In addition, cluster size varied widely, and the inflation value due to the design effect of taking clustering into account was 1.66 when using the intraclass correlation coefficient (= 0.0125) estimated by Model 2, the power was estimated as 0.898 by the “clustersampsi” procedure in Stata and our results were considered to be acceptable. Finally, we used the LOCF method to impute missing outcomes. The rates of lost to follow up for the intervention and control groups were similar (40 [5.0%] and 49 [4.9%], respectively). The main reason for non-completion overall was “having a cold”. In the control group, a significant proportion of the reasons given was “not willing” (*n* = 30). The baseline characteristics of those who had missing outcomes were not significantly different between the intervention and control groups.

## Conclusions

To date, there has been a lack of evidence from cluster RCTs regarding the effects of providing lifestyle interventions for Japanese adolescents on improving their poor SPS scores. Our trial fills this gap in the literature. Our results indicated that a lifestyle intervention program delivered at middle schools in Kumamoto, Japan improved the SPS scores of adolescents and increased the proportions of participants reporting a regular daily intake of staple foods, main dishes, and vegetables at breakfast, as well as an increased enjoyment of school life. The test population showed a moderately high adherence rate to the PADOK program, which is a positive indication of its feasibility in broader school-based home-collaborative practice. Our study provides useful information for designing lifestyle educational interventions not only for adolescents in the Kumamoto area but also for Japanese adolescents in general.

## Supporting Information

S1 CONSORT Checklist (PDF)

S1 Fig Japanese Version and English translated versions of questionnaire. (PDF)

S2 Fig Validity and reliability of the SPS score. (PDF)

S3 Fig FFQW82 (Japanese version and English translated version). (PDF)

S1 Table Mean change of the SPS score from baseline at 6 months (intervention effect on primary outcome) by multiple imputation. SPS, subjective psychosomatic symptoms; ITT/MI, Analysis by intention-to-treat principles using multiple imputation (number of imputations = 200); SE, standard error; Model 1, crude mixed model; Model 2, mixed model adjusted for baseline; Model 3, mixed model adjusted for baseline, sex, age, and BMI.
(PDF)

S2 Table Secondary analysis: Mean change of the SPS-D score from baseline at 6 months for ITT/LOCF (intervention effect on primary outcome). SPS, subjective psychosomatic symptoms; ITT/LOCF, Analysis by intention-to-treat principles performing imputation of missing data using the last observation carried forward method; SE, standard error; Model 1, crude mixed model; Model 2, mixed model adjusted for baseline; Model 3, mixed model adjusted for baseline, sex, age, and BMI.
(PDF)

S3 Table Sensitivity analysis: Mean change of the SPS and SPS-D scores from baseline at 6 months (intervention effect on primary outcome). SPS, subjective psychosomatic symptoms; PPS, Analysis by per protocol set with the complete data set; SE, standard error; Model 1, crude mixed model; Model 2, mixed model adjusted for baseline; Model 3, mixed model adjusted for baseline, sex, age, and BMI.
(PDF)

S4 Table Sensitivity analysis: Effects of lifestyle change at 6 months on resolving lifestyle factors (intervention effects on secondary outcomes; baseline-adjusted odds ratio for PPS [Models 2 & 3]). PPS, Analysis by per protocol set with the complete data set; OR (odds ratio) < 1 indicates favourable for resolving lifestyle factors; Model 2, mixed model adjusted for baseline; Model 3, mixed model adjusted for baseline, sex, age, and BMI; 1) Staple food: rice, bread, etc.; 2) Main dishes: fish, soy, eggs, meat, etc.
(PDF)
