# Supplementary material for: Effect of School-Based Home-Collaborative Lifestyle Education on Reducing Subjective Psychosomatic Symptoms in Adolescents: A Cluster Randomised Controlled Trial
Source: PLoS One. 2016 Oct 25;11(10):e0165285. doi: 10.1371/journal.pone.0165285 (PMC5079616; doi:10.1371/journal.pone.0165285)
Supplement: S1 Fig — (PDF) [file pone.0165285.s002.pdf]

S1 Fig. Japanese Version and English translated versions of questionnaire.

Japanese version

中学生の食事・生活習慣と健康質問票　（　）年（　）組（　）番　氏名（　　　　　　）

最近1ヶ月くらいのあなたの食事や生活、健康状態について、あてはまる番号を〇で囲んでください。

1	♪	朝食に、ごはん・パン・めんなどの主食を食べる。	1. いつもできる	2. 週に5～6回できる	3. 週に3～4回できる	4. 週に1～2回できる	5. まったくできない
2	♪	朝食に、卵・魚・肉・とうふ・大豆製品などの主菜を食べる。	1. いつもできる	2. 週に5～6回できる	3. 週に3～4回できる	4. 週に1～2回できる	5. まったくできない
3	♪	朝食に、野菜類を食べる。	1. いつもできる	2. 週に5～6回できる	3. 週に3～4回できる	4. 週に1～2回できる	5. まったくできない
4	♪	昼食に、卵・魚・肉・とうふ・大豆製品などの主菜を食べる。	1. いつもできる	2. 週に5～6回できる	3. 週に3～4回できる	4. 週に1～2回できる	5. まったくできない
5	♪	昼食に、野菜類を食べる。	1. いつもできる	2. 週に5～6回できる	3. 週に3～4回できる	4. 週に1～2回できる	5. まったくできない
6	♪	牛乳・ヨーグルト・豆乳などの乳製品を食べる。	1. いつもできる	2. 週に5～6回できる	3. 週に3～4回できる	4. 週に1～2回できる	5. まったくできない
7	♪	油の多い料理や食品をとり過ぎないようにする。	1. いつもできる	2. 週に5～6回できる	3. 週に3～4回できる	4. 週に1～2回できる	5. まったくできない
8	♪	夜10時以降の間食は食べない。	1. いつもできる	2. 週に5～6回できる	3. 週に3～4回できる	4. 週に1～2回できる	5. まったくできない
9	♪	運動やストレッチなど体を動かす。	1. いつもできる	2. 週に5～6回できる	3. 週に3～4回できる	4. 週に1～2回できる	5. まったくできない
10	♪	夜12時には、ぐっすり眠っている。	1. いつもできる	2. 週に5～6回できる	3. 週に3～4回できる	4. 週に1～2回できる	5. まったくできない
11	♪	睡眠時間は、6時間以上である。	1. いつもできる	2. 週に5～6回できる	3. 週に3～4回できる	4. 週に1～2回できる	5. まったくできない
12	♪	現在、自分の健康状態は	1. とてもよい	2. まあよい	3. どちらともいえない	4. あまりよくない	5. よくない
13	♫	学校生活が楽しい	1. まったく感じない	2. あまり感じない	3. ときどき感じる	4. かなり感じる	5. いつも感じる
14	♫	疲れを感じる	1. まったく感じない	2. あまり感じない	3. ときどき感じる	4. かなり感じる	5. いつも感じる
15	♫	頭痛がする	1. まったく感じない	2. あまり感じない	3. ときどき感じる	4. かなり感じる	5. いつも感じる
16	♫	身体がだるい	1. まったく感じない	2. あまり感じない	3. ときどき感じる	4. かなり感じる	5. いつも感じる
17	♫	イライラする	1. まったく感じない	2. あまり感じない	3. ときどき感じる	4. かなり感じる	5. いつも感じる
18	♫	集中力がない	1. まったく感じない	2. あまり感じない	3. ときどき感じる	4. かなり感じる	5. いつも感じる
19	♫	やる気がでない	1. まったく感じない	2. あまり感じない	3. ときどき感じる	4. かなり感じる	5. いつも感じる
20	♫	朝、すっきり起きられない	1. まったく感じない	2. あまり感じない	3. ときどき感じる	4. かなり感じる	5. いつも感じる
21	♫	胃やお腹の調子が悪い	1. まったく感じない	2. あまり感じない	3. ときどき感じる	4. かなり感じる	5. いつも感じる
22	♫	肩がこる	1. まったく感じない	2. あまり感じない	3. ときどき感じる	4. かなり感じる	5. いつも感じる

English translated version

Qestionnare of dietary intakes, lifestyle and health conditions for middle school students

Name(   ) No(   ) Class(   ) School year(   )

Please circle the number applied for your dietary intakes, lifestyle and health condition for recently one month..

1	♪	You consume staple food per breakfast.	1. Always	2. 5-6/wk	3. 3-4/wk	4. 1-2/wk	5. Not at all
2	♪	You consume main dishes per breakfast.	1. Always	2. 5-6/wk	3. 3-4/wk	4. 1-2/wk	5. Not at all
3	♪	You consume vegetables per breakfast.	1. Always	2. 5-6/wk	3. 3-4/wk	4. 1-2/wk	5. Not at all
4	♪	You consume main dishes per lunch.	1. Always	2. 5-6/wk	3. 3-4/wk	4. 1-2/wk	5. Not at all
5	♪	You consume vegetables per lunch.	1. Always	2. 5-6/wk	3. 3-4/wk	4. 1-2/wk	5. Not at all
6	♪	You consume dairy products per day.	1. Always	2. 5-6/wk	3. 3-4/wk	4. 1-2/wk	5. Not at all
7	♪	You don't consume fatty foods.	1. Always	2. 5-6/wk	3. 3-4/wk	4. 1-2/wk	5. Not at all
8	♪	You don't consume snacks after 10 PM	1. Always	2. 5-6/wk	3. 3-4/wk	4. 1-2/wk	5. Not at all
9	♪	You move your body, such as exercise and stretching..	1. Always	2. 5-6/wk	3. 3-4/wk	4. 1-2/wk	5. Not at all
10	♪	You sleep fast at 0 AM.	1. Always	2. 5-6/wk	3. 3-4/wk	4. 1-2/wk	5. Not at all
11	♪	You sleep over 6 hours.	1. Always	2. 5-6/wk	3. 3-4/wk	4. 1-2/wk	5. Not at all
12	♪	How are your health condition, now ?	1. Very good	2. Good	3. As usual	4. Fairly good	5. Not good/bad
13	♫	You feel school life is enjoyable.	1. Not at all	2. Scarecely	3. Sometimes	4. Orten	5. Always
14	♫	You feel fatigue.	1. Not at all	2. Scarecely	3. Sometimes	4. Orten	5. Always
15	♫	You feel headache.	1. Not at all	2. Scarecely	3. Sometimes	4. Orten	5. Always
16	♫	You feel lassitude	1. Not at all	2. Scarecely	3. Sometimes	4. Orten	5. Always
17	♫	You feel irritation.	1. Not at all	2. Scarecely	3. Sometimes	4. Orten	5. Always
18	♫	You feel lack of concentration.	1. Not at all	2. Scarecely	3. Sometimes	4. Orten	5. Always
19	♫	You feel lack of motivation.	1. Not at all	2. Scarecely	3. Sometimes	4. Orten	5. Always
20	♫	You feel poor ability to wake in the morning.	1. Not at all	2. Scarecely	3. Sometimes	4. Orten	5. Always
21	♫	You feel upset stomach.	1. Not at all	2. Scarecely	3. Sometimes	4. Orten	5. Always
22	♫	You feel stiff shoulders.	1. Not at all	2. Scarecely	3. Sometimes	4. Orten	5. Always
