# Supplementary material for: Effect of School-Based Home-Collaborative Lifestyle Education on Reducing Subjective Psychosomatic Symptoms in Adolescents: A Cluster Randomised Controlled Trial
Source: PLoS One. 2016 Oct 25;11(10):e0165285. doi: 10.1371/journal.pone.0165285 (PMC5079616; doi:10.1371/journal.pone.0165285)

S2 Fig. Validity and reliability of the SPS score

a) SPS score by assuming the Likert scale

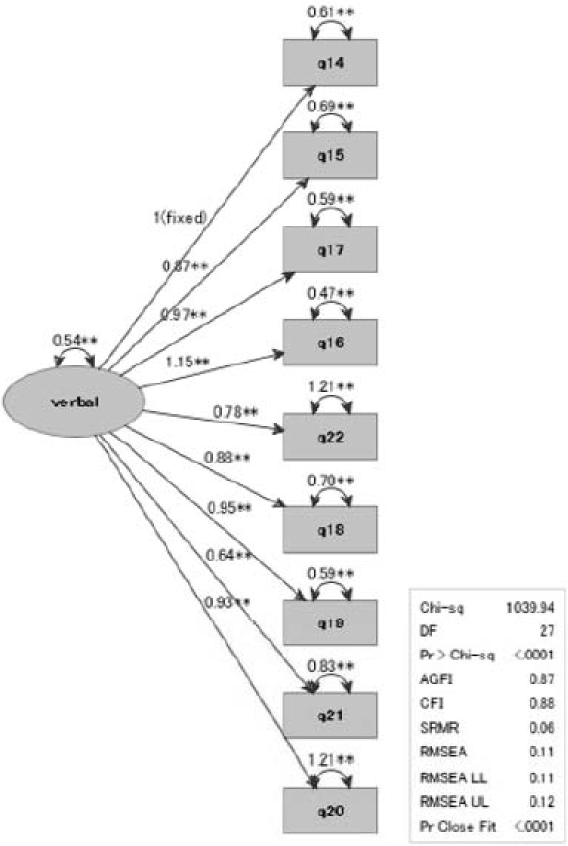

b) SPS-D score by dichotomous variable

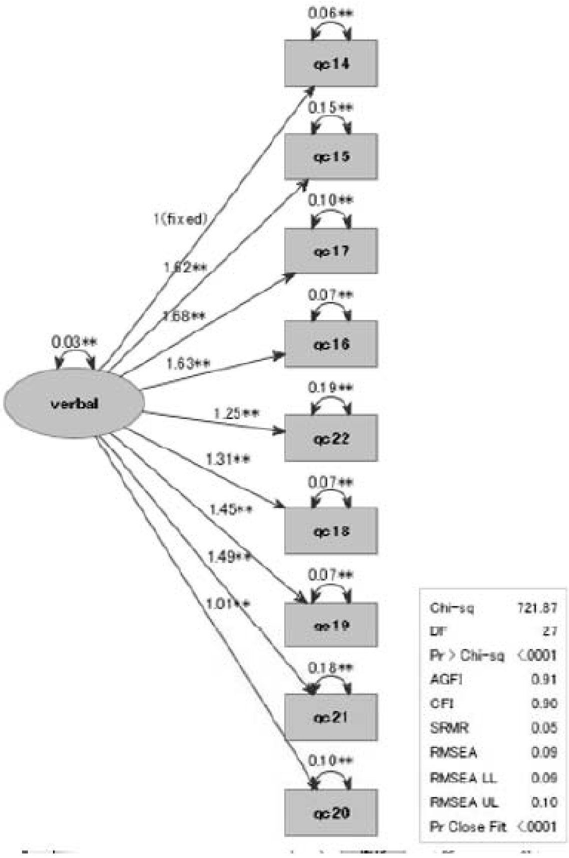

Supplement: S2 Fig — (PDF) [file pone.0165285.s003.pdf]
