# Supplementary material for: Effect of School-Based Home-Collaborative Lifestyle Education on Reducing Subjective Psychosomatic Symptoms in Adolescents: A Cluster Randomised Controlled Trial
Source: PLoS One. 2016 Oct 25;11(10):e0165285. doi: 10.1371/journal.pone.0165285 (PMC5079616; doi:10.1371/journal.pone.0165285)
Supplement: S3 Fig — (PDF) [file pone.0165285.s004.pdf]

S3 Fig. FFQW82 (Japanese version and English translated version

Japanese version

No	

FFQW82 調査票（食物摂取頻度調査）

ご回答方法

この調査は、およそ 15~20 分程度かかりますが、各項目全部にお答えいただくと、ご自身の平均的な 1 日の食事量や各栄養素の摂取量がわかります。

- この調査票のたての行には、82種類の食べ物(ごはん、塩さけ、薄切り肉など)が並んでいます。
- この調査票のよこの列には、朝食、昼食、夕食別に、各食べ物の「1 回に食べる回数」と「一回に食べる量」を示しています。
- この 1 ヶ月間でのご自身の食生活に最も近いと思われる□に「レ印」を記入してください。
- 1～82 種類の食べ物について、朝食、昼食、夕食ごとすべてにお答えください。

★ 「1 回に食べる回数」は、以下の 6 段階です。

「全く食べない」
「月に１～２回食べる」
「週に１～２回食べる」
「週に３～４回食べる」
「週に５～６回食べる」
「いつも食べる」

＊「全く食べない」食べ物にも「レ印」を記入してください。

★ 「1 回に食べる量」は、「大」、「中」、「小」とします。

「中」とは、この調査票に示した**写真の量**が目安になります。
「小」とは、「中」の**半分量**くらいです。
「大」とは、「中」の **1.5 倍量**くらいです。
「全く食べない」場合は、「1 回に食べる量」は記入しなくても結構です。

※ただし　午前中の間食は朝食の回答欄に朝食と合わせてご記入ください。
午後中の間食は昼食の回答欄に昼食と合わせてご記入ください。
夕食後に食べたものは、夕食の回答欄に合わせてご記入ください。

FFQW82　　（食物摂取頻度調査）

下記の食品は、どのくらいの頻度で食べますか。また、食べる時の1回量はどのくらいですか。
1. 食べる頻度は、「全く食べない」から「食事でいつも食べる」の6段階のいずれかの欄に「レ」点をつけてください
2. 一回に食べる量は「大」, 「中」, 「小」いずれかの欄に「レ」点を記入してください
下記に示した各食品の目安量が「中」です

「レ」点の記入例 ☑

【「中」の食品例】

類	No.	食品	No.	朝食（午前中の間食を含む） 0 全く食べない	1 月に1~2回	2 週1~2回	3 週3~4回	4 週5~6回	5 朝食にいつも	1回量 小	中	大	昼食（午後中の間食を含む） 0 全く食べない	1 月に1~2回	2 週1~2回	3 週3~4回	4 週5~6回	5 昼食にいつも	1回量 小	中	大	夕食（夜食を含む） 0 全く食べない	1 月に1~2回	2 週1~2回	3 週3~4回	4 週5~6回	5 夕食にいつも	1回量 小	中	大
穀類	1	ごはん, 赤飯, 五目ごはん 中茶わん1膳 / おにぎり / いなり寿司 3個 太巻寿司 3個 / もち 2個	1	0	1	2	3	4	5	小	中	大	0	1	2		4	5	小	中	大	0	1	2	3	4	5	小	中	大
	2	カレーライス・ハヤシライス・チャーハン など 1人前 / 弁当・定食のごはん 1人前	2	0	1	2	3	4	5	小	中	大	0	1	2	3	4	5	小	中	大	0	1	2	3	4	5	小	中	大
	3	にぎり寿司・カツ丼・親子丼・中華丼 など 1人前	3	0	1	2	3	4	5	小	中	大	0	1	2	3	4	5	小	中	大	0	1	2	3	4	5	小	中	大
	4	食パン 6枚切り1枚 / ロールパン 2個 クロワッサン 1個 / フランスパン 2cm幅3切れ / サンドイッチ 2切れ	4	0	1	2	3	4	5	小	中	大	0	1	2	3	4	5	小	中	大	0	1	2	3	4	5	小	中	大
	5	うどん・そば など 1人前 / そうめん 1.5束	5	0	1	2	3	4	5	小	中	大	0	1	2	3	4	5	小	中	大	0	1	2	3	4	5	小	中	大
	6	ラーメン・焼きそば 1人前 / インスタントラーメン / カップラーメン 1個	6	0	1	2	3	4	5	小	中	大	0	1	2	3	4	5	小	中	大	0	1	2	3	4	5	小	中	大
	7	スパゲティ・ペンネなどパスタ類	7	0	1	2	3	4	5	小	中	大	0	1	2	3	4	5	小	中	大	0	1	2	3	4	5	小	中	大
	8	じゃがいも / 里芋 中3個 / さつまいも 中1/3本 / ポテトサラダ・肉じゃが / ポテトコロッケ 2個	8	0	1	2	3	4	5	小	中	大	0	1	2	3	4	5	小	中	大	0	1	2	3	4	5	小	中	大
	9	コーンフレーク 1杯分	9	0	1	2	3	4	5	小	中	大	0	1	2	3	4	5	小	中	大	0	1	2	3	4	5	小	中	大
	10	ピザ 1人前 / ホットケーキ（パンケーキ）2枚分 / お好み焼き 1枚分 たこ焼き 8個 / マカロニサラダ 小鉢1杯	10	0	1	2	3	4	5	小	中	大	0	1	2	3	4	5	小	中	大	0	1	2	3	4	5	小	中	大
	11	ぎょうざ 5個 / しゅうまい 大3個 / 春巻き 2本 / これらの皮・片栗粉など	11	0	1	2	3	4	5	小	中	大	0	1	2	3	4	5	小	中	大	0	1	2	3	4	5	小	中	大
魚介類	12	塩さけ 1切れ / あじ開き 1枚 / ほっけ開き 1/2枚 / みそ漬け魚・かす漬け魚 1切れ	12	0	1	2	3	4	5	小	中	大	0	1	2	3	4	5	小	中	大	0	1	2	3	4	5	小	中	大
	13	あじたたき 1尾分 / たい・ひらめ刺身 5切れ / めかじき・たら かれい など 1切れ	13	0	1	2	3	4	5	小	中	大	0	1	2	3	4	5	小	中	大	0	1	2	3	4	5	小	中	大
	14	さんま 1/2尾 / さば 1/4尾 / ぶり・銀だら・さわら 1切れ / まぐろ・かつお・はまち 刺身 4切 / いわし 1尾	14	0	1	2	3	4	5	小	中	大	0	1	2	3	4	5	小	中	大	0	1	2	3	4	5	小	中	大
	15	えび / いか 1/2杯 / まだこ 足1本分	15	0	1	2	3	4	5	小	中	大	0	1	2	3	4	5	小	中	大	0	1	2	3	4	5	小	中	大
	16	ほたて貝柱 2個 / あさり 10～12個 / しじみ味噌汁 / カキ 3個	16	0	1	2	3	4	5	小	中	大	0	1	2	3	4	5	小	中	大	0	1	2	3	4	5	小	中	大
	17	しらす干し / たらこ 1/2本 いくら 大さじ2 / 塩辛 / 貝・のり等佃煮 大さじ1杯	17	0	1	2	3	4	5	小	中	大	0	1	2	3	4	5	小	中	大	0	1	2	3	4	5	小	中	大
	18	かまぼこ / 焼きちくわ / さつまあげ 1枚	18	0	1	2	3	4	5	小	中	大	0	1	2	3	4	5	小	中	大	0	1	2	3	4	5	小	中	大
	19	小)ツナ缶 1/2缶 / さば味噌煮缶 1/3缶 / さんまかば焼き缶 いわし味噌煮缶 1/2缶	19	0	1	2	3	4	5	小	中	大	0	1	2	3	4	5	小	中	大	0	1	2	3	4	5	小	中	大
肉類	20	しょうが焼き 3枚 / 豚カツ 1/2枚 / 肉煮込み 小鉢1杯 / 肉野菜炒め 1皿 / 串カツ 2本	20	0	1	2	3	4	5	小	中	大	0	1	2	3	4	5	小	中	大	0	1	2	3	4	5	小	中	大
	21	鶏もも肉, / からあげ 3個 / 焼き鳥 2本 / 筑前煮 小鉢1杯 / 煮物、野菜炒め、スープなどに入る肉やひき肉も合わせて、	21	0	1	2	3	4	5	小	中	大	0	1	2	3	4	5	小	中	大	0	1	2	3	4	5	小	中	大
	22	ステーキ 1/2枚 / 牛丼 1人前 / ビーフシチュー 1人前 / 牛肉薄切り 2枚	22	0	1	2	3	4	5	小	中	大	0	1	2	3	4	5	小	中	大	0	1	2	3	4	5	小	中	大
	23	ハンバーグ 1個 / ミートボール 3個 / メンチカツ 1個 / ミートソース・その他 ひき肉料理 1人前	23	0	1	2	3	4	5	小	中	大	0	1	2	3	4	5	小	中	大	0	1	2	3	4	5	小	中	大
	24	ハム 2枚 / ウインナー 2本 / ベーコン 2枚	24	0	1	2	3	4	5	小	中	大	0	1	2	3	4	5	小	中	大	0	1	2	3	4	5	小	中	大
	25	レバー・タン・ハツなど 2串 / もつ煮 1人前	25	0	1	2	3	4	5	小	中	大	0	1	2	3	4	5	小	中	大	0	1	2	3	4	5	小	中	大

下記の食品は、どのくらいの頻度で食べますか。また、食べる時の1回量はどのくらいですか。

1. 食べる頻度は、「全く食べない」から「食事でいつも食べる」の6段階のいずれかの欄に「レ」点をつけてください

2. 一回に食べる量は「大」,「中」,「小」いずれかの欄に「レ」点を記入してください

下記に示した各食品の目安量が「中」です

「レ」点の記入例 ☑

【「中」の食品例】

分類	No.	食品例	No.	朝食（午前中の間食を含む）						1回量			昼食（午後中の間食を含む）						1回量			夕食（夜食を含む）						1回量		
				0 全く食べない	1 月に1～2回	2 週1～2回	3 週3～4回	4 週5～6回	5 朝食にいつも	小	中	大	0 全く食べない	1 月に1～2回	2 週1～2回	3 週3～4回	4 週5～6回	5 昼食にいつも	小	中	大	0 全く食べない	1 月に1～2回	2 週1～2回	3 週3～4回	4 週5～6回	5 夕食にいつも	小	中	大
卵	26	たまご 1個 / 卵豆腐 1個(100g) / 茶碗蒸し 大1個	26	0	1	2	3	4	5	小	中	大	0	1	2	3	4	5	小	中	大	0	1	2	3	4	5	小	中	大
ﾁｰｽﾞ	27	プロセスチーズ カマンベールチーズ 1切れ / スライスチーズ 1枚 / 6Pチーズ 1個 / ピザ用チーズ 1切れ分	27	0	1	2	3	4	5	小	中	大	0	1	2	3	4	5	小	中	大	0	1	2	3	4	5	小	中	大
大豆類	28	とうふ 1/2丁 / 凍りとうふ 1個 / 煮物、味噌汁などに入る豆腐や油あげ、厚揚げ、がんもも合わせて、	28	0	1	2	3	4	5	小	中	大	0	1	2	3	4	5	小	中	大	0	1	2	3	4	5	小	中	大
	29	納豆 小1個 / ゆで大豆 大さじ3 / えだまめ 1カップ / 五目大豆	29	0	1	2	3	4	5	小	中	大	0	1	2	3	4	5	小	中	大	0	1	2	3	4	5	小	中	大
	30	油あげ 1/4枚	30	0	1	2	3	4	5	小	中	大	0	1	2	3	4	5	小	中	大	0	1	2	3	4	5	小	中	大
	31	厚あげ 1/4枚 / がんも 中1/2個	31	0	1	2	3	4	5	小	中	大	0	1	2	3	4	5	小	中	大	0	1	2	3	4	5	小	中	大
野菜・海そう類	32	サラダ 小鉢 1杯（レタス・きゅうり・トマト） / せんキャベツ 小鉢1杯 / 大根サラダ 小鉢 1杯 / きゅうり酢の物 小鉢 1杯	32	0	1	2	3	4	5	小	中	大	0	1	2	3	4	5	小	中	大	0	1	2	3	4	5	小	中	大
	33	青菜おひたし 小鉢 1杯 / ブロッコリー 5ふさ / いんげん・もやし 小鉢1杯	33	0	1	2	3	4	5	小	中	大	0	1	2	3	4	5	小	中	大	0	1	2	3	4	5	小	中	大
	34	野菜いため / 八宝菜 中皿1杯	34	0	1	2	3	4	5	小	中	大	0	1	2	3	4	5	小	中	大	0	1	2	3	4	5	小	中	大
	35	野菜煮物 小鉢 1杯（大根・人参・ごぼう） / きんぴらごぼう 小鉢1杯 / おでん・ふろふき大根 大根1切れ	35	0	1	2	3	4	5	小	中	大	0	1	2	3	4	5	小	中	大	0	1	2	3	4	5	小	中	大
	36	かぼちゃ煮物 / かぼちゃサラダ	36	0	1	2	3	4	5	小	中	大	0	1	2	3	4	5	小	中	大	0	1	2	3	4	5	小	中	大
	37	みそ汁や野菜スープ （キャベツ・大根・人参・玉ねぎ・長ねぎ など） 1杯分	37	0	1	2	3	4	5	小	中	大	0	1	2	3	4	5	小	中	大	0	1	2	3	4	5	小	中	大
	38	しいたけ, しめじ, えのき, エリンギなど 小鉢1杯 / なめこ 大さじ3杯分	38	0	1	2	3	4	5	小	中	大	0	1	2	3	4	5	小	中	大	0	1	2	3	4	5	小	中	大
	39	こんにゃく 1/4枚 / しらたき 1/3玉（袋）	39	0	1	2	3	4	5	小	中	大	0	1	2	3	4	5	小	中	大	0	1	2	3	4	5	小	中	大
	40	塩漬け, ぬか漬け 5切れ / たくあん漬け 3切れ / 野沢菜, 高菜, キムチ, 福神漬 小皿1杯	40	0	1	2	3	4	5	小	中	大	0	1	2	3	4	5	小	中	大	0	1	2	3	4	5	小	中	大
	41	ひじき煮物 / 生わかめ・めかぶ・もずく	41	0	1	2	3	4	5	小	中	大	0	1	2	3	4	5	小	中	大	0	1	2	3	4	5	小	中	大
乳類	42	普通牛乳 コップ1杯	42	0	1	2	3	4	5	小	中	大	0	1	2	3	4	5	小	中	大	0	1	2	3	4	5	小	中	大
	43	低脂肪乳 コップ1杯 / スキムミルク 大さじ5杯	43	0	1	2	3	4	5	小	中	大	0	1	2	3	4	5	小	中	大	0	1	2	3	4	5	小	中	大
	44	ヨーグルト 小鉢1杯(100g) / カップ入り ヨーグルト1個 / 飲むヨーグルト	44	0	1	2	3	4	5	小	中	大	0	1	2	3	4	5	小	中	大	0	1	2	3	4	5	小	中	大
果物類	45	みかん 中2個 / オレンジ・いよかん・夏みかん 1個 / グレープフルーツ 1/2個	45	0	1	2	3	4	5	小	中	大	0	1	2	3	4	5	小	中	大	0	1	2	3	4	5	小	中	大
	46	バナナ 1本 / りんご, なし, 柿 1/2個 / すいか / いちご, ぶどう 両手1杯分	46	0	1	2	3	4	5	小	中	大	0	1	2	3	4	5	小	中	大	0	1	2	3	4	5	小	中	大
飲料	47	豆乳 コップ1杯	47	0	1	2	3	4	5	小	中	大	0	1	2	3	4	5	小	中	大	0	1	2	3	4	5	小	中	大
	48	野菜ジュース（人参・ミックスなど） コップ1杯	48	0	1	2	3	4	5	小	中	大	0	1	2	3	4	5	小	中	大	0	1	2	3	4	5	小	中	大
	49	果物ジュース（オレンジ・グレープフルーツ・りんご・ぶどう・ミックスなど） コップ1杯	49	0	1	2	3	4	5	小	中	大	0	1	2	3	4	5	小	中	大	0	1	2	3	4	5	小	中	大

下記の食品は、どのくらいの頻度で食べますか。また、食べる時の1回量はどのくらいですか。

1. 食べる頻度は、「全く食べない」から「食事でいつも食べる」の6段階のいずれかの欄に「レ」点をつけてください

2. 一回に食べる量は「大」,「中」,「小」いずれかの欄に「レ」点を記入してください

下記に示した各食品の目安量が「中」です

「レ」点の記入例 ☑

【「中」の食品例】

分類	No.	「中」の食品例
アルコール類	50	缶ビール500ml 1缶
	51	日本酒 1合
	52	ワイン ワイングラス1杯
	53	焼酎ストレート グラス1/4杯　缶入りサワー 1/2缶 サワー 大グラス1/2杯　ウイスキー シングル1杯　梅酒 グラス1杯
茶類	54	せん茶，ほうじ茶，番茶，ウーロン茶，紅茶 など　コップ1杯
	55	コーヒー(ドリップ・インスタント) カップ1杯　缶コーヒー(無糖) 1本 アイスコーヒー(無糖) コップ 1杯　＊糖分入りコーヒーは除く
嗜好品類	56	スポーツ飲料 グラス1杯　炭酸飲料 グラス1杯　果汁入り飲料 グラス1杯　乳酸飲料 小1本　糖分入りコーヒー 1缶
	57	うずら豆など 小皿1杯　こんぶ豆 小皿1杯　ゆであずき 大さじ1杯強
	58	菓子パン：あんぱん，クリームパン，ジャムパン，メロンパン，チョコロネ，カレーパン など 1個
	59	ショートケーキ・チョコレートケーキ パイ・タルト・ドーナツ など 1個　パウンドケーキ 3cm　シュークリーム 1個
	60	まんじゅう 小1個 ようかん 1cm幅　カステラ 1cm幅　どらやき・もなか・大福など 1/3個
	61	プリン　ゼリー・ババロア 1個　シャーベット 1個
	62	チョコレート 3かけら　チョコ菓子 片手1杯分　シャーベット 1個
	63	揚げせんべい 2枚　かりんとう(黒・白) 3～5本　ポテトチップス・ポップコーン コーンスナック 片手1杯分
	64	あめ，ドロップ 2個　キャラメル 2個　バタースカッチ 2個　ガム 4枚
	65	カップアイスクリーム 1個　アイスバー 1本　ソフトクリーム 1個
	66	かた焼きせんべい 1枚　2枚入りせんべい　かきもち 2枚
	67	クッキー 2枚　サブレ 1枚　ビスケット 3枚
汁もの	68	みそ汁 1杯分
	69	すまし汁，野菜スープ，けんちん汁 など 1杯分
	70	ポタージュ （カップスープ，インスタントスープを含む）1人前
使った料理さとうやしょうゆ	71	さとう，はちみつ，ジャム など 小さじ1　スティックシュガー(3g) 1本
	72	煮物(かぼちゃ，いも類)，きんぴらごぼう など 小鉢1杯
	73	「おひたし」，「おさしみ」などに使うしょうゆやポン酢 小さじ1
	74	ソース，ケチャップ 大さじ1

No.	朝食（午前中の間食を含む） 0 全く食べない	1 月に1～2回	2 週1～2回	3 週3～4回	4 週5～6回	5 朝食にいつも	1回量 小	中	大	昼食（午後中の間食を含む） 0 全く食べない	1 月に1～2回	2 週1～2回	3 週3～4回	4 週5～6回	5 昼食にいつも	1回量 小	中	大	夕食（夜食を含む） 0 全く食べない	1 月に1～2回	2 週1～2回	3 週3～4回	4 週5～6回	5 夕食にいつも	1回量 小	中	大
50	0	1	2	3	4	5	小	中	大	0	1	2		4	5	小	中	大	0	1	2	3	4	5	小	中	大
51	0	1	2	3	4	5	小	中	大	0	1	2	3	4	5	小	中	大	0	1	2	3	4	5	小	中	大
52	0	1	2	3	4	5	小	中	大	0	1	2	3	4	5	小	中	大	0	1	2	3	4	5	小	中	大
53	0	1	2	3	4	5	小	中	大	0	1	2	3	4	5	小	中	大	0	1	2	3	4	5	小	中	大
54	0	1	2	3	4	5	小	中	大	0	1	2	3	4	5	小	中	大	0	1	2	3	4	5	小	中	大
55	0	1	2	3	4	5	小	中	大	0	1	2	3	4	5	小	中	大	0	1	2	3	4	5	小	中	大
56	0	1	2	3	4	5	小	中	大	0	1	2	3	4	5	小	中	大	0	1	2	3	4	5	小	中	大
57	0	1	2	3	4	5	小	中	大	0	1	2	3	4	5	小	中	大	0	1	2	3	4	5	小	中	大
58	0	1	2	3	4	5	小	中	大	0	1	2	3	4	5	小	中	大	0	1	2	3	4	5	小	中	大
59	0	1	2	3	4	5	小	中	大	0	1	2	3	4	5	小	中	大	0	1	2	3	4	5	小	中	大
60	0	1	2	3	4	5	小	中	大	0	1	2	3	4	5	小	中	大	0	1	2	3	4	5	小	中	大
61	0	1	2	3	4	5	小	中	大	0	1	2	3	4	5	小	中	大	0	1	2	3	4	5	小	中	大
62	0	1	2	3	4	5	小	中	大	0	1	2	3	4	5	小	中	大	0	1	2	3	4	5	小	中	大
63	0	1	2	3	4	5	小	中	大	0	1	2	3	4	5	小	中	大	0	1	2	3	4	5	小	中	大
64	0	1	2	3	4	5	小	中	大	0	1	2	3	4	5	小	中	大	0	1	2	3	4	5	小	中	大
65	0	1	2	3	4	5	小	中	大	0	1	2	3	4	5	小	中	大	0	1	2	3	4	5	小	中	大
66	0	1	2	3	4	5	小	中	大	0	1	2	3	4	5	小	中	大	0	1	2	3	4	5	小	中	大
67	0	1	2	3	4	5	小	中	大	0	1	2	3	4	5	小	中	大	0	1	2	3	4	5	小	中	大
68	0	1	2	3	4	5	小	中	大	0	1	2	3	4	5	小	中	大	0	1	2	3	4	5	小	中	大
69	0	1	2	3	4	5	小	中	大	0	1	2	3	4	5	小	中	大	0	1	2	3	4	5	小	中	大
70	0	1	2	3	4	5	小	中	大	0	1	2	3	4	5	小	中	大	0	1	2	3	4	5	小	中	大
71	0	1	2	3	4	5	小	中	大	0	1	2	3	4	5	小	中	大	0	1	2	3	4	5	小	中	大
72	0	1	2	3	4	5	小	中	大	0	1	2	3	4	5	小	中	大	0	1	2	3	4	5	小	中	大
73	0	1	2	3	4	5	小	中	大	0	1	2	3	4	5	小	中	大	0	1	2	3	4	5	小	中	大
74	0	1	2	3	4	5	小	中	大	0	1	2	3	4	5	小	中	大	0	1	2	3	4	5	小	中	大

＊　以下の項目は**油類の使用頻度についてお聞きします**ので、
すでにお答えになった料理についても、再度、ご回答願います。

		【「中」の食品例】		朝食（午前中の間食を含む）									昼食（午後中の間食を含む）									夕食（夜食を含む）								
				0 全く食べない	1 月に1～2回	2 週1～2回	3 週3～4回	4 週5～6回	5 朝食にいつも	1回量 小	中	大	0 全く食べない	1 月に1～2回	2 週1～2回	3 週3～4回	4 週5～6回	5 昼食にいつも	1回量 小	中	大	0 全く食べない	1 月に1～2回	2 週1～2回	3 週3～4回	4 週5～6回	5 夕食にいつも	1回量 小	中	大
油脂類	75	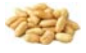 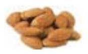 ピーナッツ・アーモンド・カシューナッツ　など　片手1杯 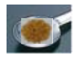 ごま 大さじ1	75	0	1	2	3	4	5	小	中	大	0	1	2		4	5	小	中	大	0	1	2	3	4	5	小	中	大
	76	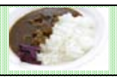 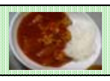 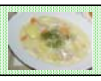 カレーライス・ハヤシライス・シチュー　など　1人前	76	0	1	2	3	4	5	小	中	大	0	1	2	3	4	5	小	中	大	0	1	2	3	4	5	小	中	大
	77	フレンチドレッシング・クリーミードレッシング・マヨネーズ など　大さじ1 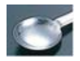 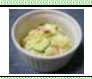 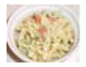 ポテトサラダ・マカロニサラダなど 小鉢1杯	77	0	1	2	3	4	5	小	中	大	0	1	2	3	4	5	小	中	大	0	1	2	3	4	5	小	中	大
	78	ノンオイルドレッシング　大さじ1 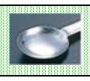	78	0	1	2	3	4	5	小	中	大	0	1	2	3	4	5	小	中	大	0	1	2	3	4	5	小	中	大
	79	バター・マーガリン　大さじ1 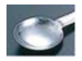 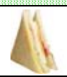 サンドイッチ 2切れ 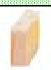 固形バター・マーガリン 1切れ	79	0	1	2	3	4	5	小	中	大	0	1	2	3	4	5	小	中	大	0	1	2	3	4	5	小	中	大
	80	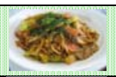 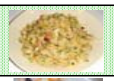 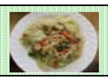 焼きそば，チャーハン，ビーフン　1人前	80	0	1	2	3	4	5	小	中	大	0	1	2	3	4	5	小	中	大	0	1	2	3	4	5	小	中	大
	81	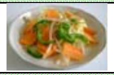 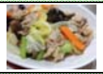 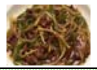 野菜炒め　中皿1杯分，中国炒めもの　1人前	81	0	1	2	3	4	5	小	中	大	0	1	2	3	4	5	小	中	大	0	1	2	3	4	5	小	中	大
	82	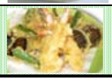 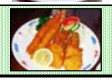 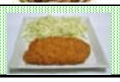 天ぷら，フライ，カツ　1人前 コロッケ　2個 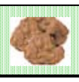 から揚げ 4個 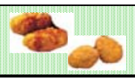 冷凍食品 あげ物2個	82	0	1	2	3	4	5	小	中	大	0	1	2	3	4	5	小	中	大	0	1	2	3	4	5	小	中	大

★ご記入お願いします
【性別】　男　・　女
【年齢】　（　　　　　）歳
【身長】　（　　　　　）cm
【体重】　（　　　　　）kg
【日常生活】一週間の平均的な日常生活を教えてください。どれか一つに○印をつけてください。
1．座っている仕事が多い・家にいることが多い
2．立ってする仕事や歩き回る仕事が多い、または20分以上の運動等を週に3回以上行う
3．頻繁に休まないとできない仕事が多い、または毎日1時間以上の運動を行う

English translated version

FFQW82(Semi-Quantative Food Frequency Questionnaire W82)

During the past a month, how frequency did you eat a serving of each of the foods listed here?
① The response 6 categories for the frequency: Never, 1～2 per month, 1～2 per week, 3～4 per week, 5～6 per week, Every time
Please mark only one ✓ for frequency
② The response 3 categories for the portion size:
Small (S); less than 0.5 portion size, Medium (M); 1 portion size, Large (L); more than 1.5 portion size
You can see "Medium; 1 portion size" as following photograph of each food.
Please mark only one ✓for portion size. If you didn't eat the food, no ✓mark.

Group	No.	"Medium" 1 portion size (for example)	Frequency	Breakfast (including the morning snack)									Lunch (including the afternoon snack)									Dinner (including the night snack)								
				0 Never	1 1～2 per month	2 1～2 per week	3 3～4 per week	4 5～6 per week	5 Everytime	portion size S	M	L	0 Never	1 1～2 per month	2 1～2 per week	3 3～4 per week	4 5～6 per week	5 Everytime	portion size S	M	L	0 Never	1 1～2 per month	2 1～2 per week	3 3～4 per week	4 5～6 per week	5 Everytime	portion size S	M	L
Grains group (included Potatos)	1	Rice, 1 cup medium; RICE BALL 1.5 pieces; INARI-SUSHI or FUTOMAKI-SUSHI, 3 pieces; Rice cake, 2 pieces	1	0	1	2	3	4	5				0	1	2	3	4	5				0	1	2	3	4	5			
	2	Curry and rice or Chinese-style fried rice, 1 portion size; Rice of Lunch box or Set meal	2	0	1	2	3	4	5				0	1	2	3	4	5				0	1	2	3	4	5			
	3	NIGIRIZUSHI or KATHUDON, or OYAKODON, 1 portion size	3	0	1	2	3	4	5				0	1	2	3	4	5				0	1	2	3	4	5			
	4	White bread , one of 6-cutting; Soft rolls, 2 pieces; French bread, 3 pieces; Sandwich, 2 cuts	4	0	1	2	3	4	5				0	1	2	3	4	5				0	1	2	3	4	5			
	5	UDON or SOBA, 1 portion size; SOMEN , 1 portion size	5	0	1	2	3	4	5				0	1	2	3	4	5				0	1	2	3	4	5			
	6	Chinese noodles or YAKISOBA,; Instant chinese noodles, 1 portion size; Instant chinese noodles packed in cups,1 portion	6	0	1	2	3	4	5				0	1	2	3	4	5				0	1	2	3	4	5			
	7	Pasta(Spaghetti and Penne), 1 portion size	7	0	1	2	3	4	5				0	1	2	3	4	5				0	1	2	3	4	5			
	8	Potatoes, 1 portion size; Taro:SATOIMO 3 of mideum size; Sweet potato, 1/3 of mideum size; NIKUZYAGA, 1 portion; Potato salad orCroquettes,	8	0	1	2	3	4	5				0	1	2	3	4	5				0	1	2	3	4	5			
	9	Cornflakes, 1 portion size	9	0	1	2	3	4	5				0	1	2	3	4	5				0	1	2	3	4	5			
	10	Pizza 1 portion size; Pancake 2 portion size; OKONOMIYAKI, 1 sheet or TAKOYAKI, 8 items; Macaroni salad 1 cup small bowl	10	0	1	2	3	4	5				0	1	2	3	4	5				0	1	2	3	4	5			
	11	JIAOZIChibese meat dumpling), 5 items; SHUMAI(Chinese meat dumpling), 3 large items; HARUMAKI, 2 items; Outer steamed wheat dough of JIAOZI and SHUMAI or	11	0	1	2	3	4	5				0	1	2	3	4	5				0	1	2	3	4	5			
Seafood group	12	SHIOZAKE (salted salmon) 1 piece; Horse mackerel ,HIRAKIBOSHI; Atka mackerel, HIRAKIBOSHI (salted)	12	0	1	2	3	4	5				0	1	2	3	4	5				0	1	2	3	4	5			
	13	Horse ackerel ,TATAKI, 1; Sea bream or olive flounder (raw),SASHIMI, 5; Swordfish·Cod·Righteye flounder	13	0	1	2	3	4	5				0	1	2	3	4	5				0	1	2	3	4	5			
	14	Pacific saury, 1/2 portion; Mackerel, 1/4 portion; Yellowtail·Japanese Spanish mackerel,1 piece; Sardine, 1 portion; SASHIMI, 4 pieces	14	0	1	2	3	4	5				0	1	2	3	4	5				0	1	2	3	4	5			
	15	Kuruma prawn, 4 portion size; Japanese common squid, 1/4 portion size; Common octopus, 1 portion size	15	0	1	2	3	4	5				0	1	2	3	4	5				0	1	2	3	4	5			
	16	Giant ezo-scallop, adductor muscle, 2; Short-necked clam, 10～12 pieces; Miso soup of Japanese corbicula; Pacific oyster 3 pieces	16	0	1	2	3	4	5				0	1	2	3	4	5				0	1	2	3	4	5			
	17	Sardine, SHIRASUBOSHI (boiled and dried whitebait), 2 spoons; Walleye pollock, TARAKO (salted) 1/2; Chum salmon, IKURA 2 large spoons; Algae, TSUKUDANI (simmered in soy sauce and sugar),1 large	17	0	1	2	3	4	5				0	1	2	3	4	5				0	1	2	3	4	5			
	18	Surimi products, MUSHI— KAMABOKO,; Surimi products, YAKI-CHIKUWA, 1 piece; Surimi products, SATSUMA-AGE, 1piece	18	0	1	2	3	4	5				0	1	2	3	4	5				0	1	2	3	4	5			
	19	Tuna, canned products (small),1/2 can; Mackerel, canned products,1/3 can; Pacific saury(canned products) or Japanese pilchard(canned products),1/2	19	0	1	2	3	4	5				0	1	2	3	4	5				0	1	2	3	4	5			
Meat group	20	Pork,SHOGAYAK I,3 pieces; Pork,TONKATU, 1/2 pieces; Pork stew, 1 cup small; Stir-fried pork vegetables,	20	0	1	2	3	4	5				0	1	2	3	4	5				0	1	2	3	4	5			
	21	Chicken(thigh,breast) 1/2 piece; Fried chicken, 3 pieces; Chicken,YAKITORI, 2 skewers; Chicken stew, 1 cup small bowl	21	0	1	2	3	4	5				0	1	2	3	4	5				0	1	2	3	4	5			
	22	Beef steak, 1/2 piece; Beef,GYUDON, 1 Beef Stew; Beef Stew, 1 portion; Beef thin slice, 2 pieces	22	0	1	2	3	4	5				0	1	2	3	4	5				0	1	2	3	4	5			
	23	Hamburger, 1 portion size; Meatball ,3; Mince cutlet, 1 piece; Meat sauce and other mince dish, 1 portion size	23	0	1	2	3	4	5				0	1	2	3	4	5				0	1	2	3	4	5			
	24	Pork ham(loin); Pork sausage(Vienna),; Pork bacon 2 pieces; Please think about meat and minced meat such as stew, stir-fried vegetables,soup.	24	0	1	2	3	4	5				0	1	2	3	4	5				0	1	2	3	4	5			
	25	YAKITORI(liver,tongue,heart); MOTUNI, 1 portion size	25	0	1	2	3	4	5				0	1	2	3	4	5				0	1	2	3	4	5			

FFQW82(Semi-Quantative Food Frequency Questionnaire W82)

During the past a month, how frequency did you eat a serving of each of the foods listed here?
① The response 6 categories for the frequency: Never, 1~2 per month, 1~2 per week, 3~4 per week, 5~6 per week, Every time
Please mark only one ✓ for frequency
② The response 3 categories for the portion size:
Small (S); less than 0.5 portion size, Medium (M); 1 portion size, Large (L); more than 1.5 portion size
You can see "Medium; 1 portion size" as following photograph of each food.
Please mark only one ✓ for portion size. If you didn't eat the food, no ✓ mark.

Group	No.	"Medium" 1 portion size (for example)	Frequency	Breakfast (including the morning snack)									Lunch (including the afternoon snack)									Dinner (including the night snack)								
				0 Never	1 1~2 per month	2 1~2 per week	3 3~4 per week	4 5~6 per week	5 Everytime	portion size S	M	L	0 Never	1 1~2 per month	2 1~2 per week	3 3~4 per week	4 5~6 per week	5 Everytime	portion size S	M	L	0 Never	1 1~2 per month	2 1~2 per week	3 3~4 per week	4 5~6 per week	5 Everytime	portion size S	M	L
Egg	26	Egg 1 egg; Eggs, hen, TAMAGO-DOFU,; Eggs, CHAWANMUSHI, 1 portion size	26	0	1	2	3	4	5				0	1	2	3	4	5				0	1	2	3	4	5			
Cheese	27	Cheeses, processed・Camembert 1 slice; slice cheese 1 slice; 6P cheese 1piece; Cheese for pizza	27	0	1	2	3	4	5				0	1	2	3	4	5				0	1	2	3	4	5			
Soybeans group	28	Soybeans, TOFU,; Freeze dried tofu	28	0	1	2	3	4	5				0	1	2	3	4	5				0	1	2	3	4	5			
	29	Soybeans, NATTO,; Boiled soybeans,; Soybeans, EDAMAME,; Soybeans, GOMOKUMAME,	29	0	1	2	3	4	5				0	1	2	3	4	5				0	1	2	3	4	5			
	30	Soybeans, tofu, ABURA-AGE, 1/4 size	30	0	1	2	3	4	5				0	1	2	3	4	5				0	1	2	3	4	5			
	31	Fried slices of drained tofu,ATUAGE, 1/4 cut size; Soybeans, tofu, GANMO, half size	31	0	1	2	3	4	5				0	1	2	3	4	5				0	1	2	3	4	5			
Vegetables・Algaes group	32	Vegetable salad(Lettuce・cucumber・tomatos),1 portion; Shredded cabbage,; Radish salad, 1 portion size; Cucumber vinegar, 1 portion size	32	0	1	2	3	4	5				0	1	2	3	4	5				0	1	2	3	4	5			
	33	Spinach boiled, 1 portion size; Broccoli boiled, 1 portion size; Kidney beans boiled・Bean sprouts boiled,	33	0	1	2	3	4	5				0	1	2	3	4	5				0	1	2	3	4	5			
	34	Fried vegetables, 1 portion size; Chop suey, 1 portion size	34	0	1	2	3	4	5				0	1	2	3	4	5				0	1	2	3	4	5			
	35	Boiled vegetable(Japanese radishes, Daikon・carrot・Edible burdock), 1; Kinpira burdock,; ODEN・FUROFUKI,radish,	35	0	1	2	3	4	5				0	1	2	3	4	5				0	1	2	3	4	5			
	36	Boiled pumpkin, 1 portion size; Salad pumpkin, 1 portion size	36	0	1	2	3	4	5				0	1	2	3	4	5				0	1	2	3	4	5			
	37	Miso soup・vegitable soup(cabbage・radish・carrot・onion・Welsh onions, NEBUKA-NEGI), 1 portion size	37	0	1	2	3	4	5				0	1	2	3	4	5				0	1	2	3	4	5			
	38	Mushrooms, SHIITAKE・HATAKESHIMEJI・winter mushrooms・king oyster mushrooms, 1; NAMEKO, 3 table spoons	38	0	1	2	3	4	5				0	1	2	3	4	5				0	1	2	3	4	5			
	39	KONJAC, 1/4 cut; SHIRATAKI, 1/3 ball	39	0	1	2	3	4	5				0	1	2	3	4	5				0	1	2	3	4	5			
	40	Salted vegetables・pickled in salty rice bran paste vegetables, 5; TAKUAN, 3 pieces; Salted NOZAWANA, TAKANA, KIMCHI, HUKUJIN, 1 portion size	40	0	1	2	3	4	5				0	1	2	3	4	5				0	1	2	3	4	5			
	41	HIJIKI simmered, 1 portion size; Raw seaweed・Turnips・MOZUKU, 1 portion size	41	0	1	2	3	4	5				0	1	2	3	4	5				0	1	2	3	4	5			
Milk group	42	Whole milk, one glass	42	0	1	2	3	4	5				0	1	2	3	4	5				0	1	2	3	4	5			
	43	Low-fat milk, one galass; Skim milk, 5 table spoons	43	0	1	2	3	4	5				0	1	2	3	4	5				0	1	2	3	4	5			
	44	Yoghurt, one cup(100g); cup yogurt, one cup; yogurt drink, one galass	44	0	1	2	3	4	5				0	1	2	3	4	5				0	1	2	3	4	5			
Fruit grou	45	Satsuma mandarins 2 mandarins; Orange・citrus, IYO・citrus, NATSUDAIDAI, two; Grapefruit grapefruit half	45	0	1	2	3	4	5				0	1	2	3	4	5				0	1	2	3	4	5			
	46	Banana. one; Apple・pear・persimmon. half an fruit; Watermelon. 2 slices; Strawberry・grape Both hands full	46	0	1	2	3	4	5				0	1	2	3	4	5				0	1	2	3	4	5			
beverage	47	Soy milk, 1 glass	47	0	1	2	3	4	5				0	1	2	3	4	5				0	1	2	3	4	5			
	48	Vegetable juice(carrot) 1glass	48	0	1	2	3	4	5				0	1	2	3	4	5				0	1	2	3	4	5			
	49	Fruit juice(orange・grapefruit・apple・grape・mix juice) 1 cup	49	0	1	2	3	4	5				0	1	2	3	4	5				0	1	2	3	4	5			

FFQW82(Semi-Quantative Food Frequency Questionnaire W82)

During the past a month, how frequency did you eat a serving of each of the foods listed here?
① The response 6 categories for the frequency: Never, 1～2 per month, 1～2 per week, 3～4 per week, 5～6 per week, Every time
Please mark only one ✓ for frequency
② The response 3 categories for the portion size:
Small (S); less than 0.5 portion size, Medium (M); 1 portion size, Large (L); more than 1.5 portion size
You can see "Medium; 1 portion size" as following photograph of each food.
Please mark only one ✓for portion size. If you didn't eat the food, no ✓mark.

"Medium" 1 portion size (for example)

			Frequency	Breakfast (including the morning snack)									Lunch (including the afternoon snack)									Dinner (including the night snack)								
				0 Never	1 1～2 per month	2 1～2 per week	3 3～4 per week	4 5～6 per week	5 Everytime	portion size S	M	L	0 Never	1 1～2 per month	2 1～2 per week	3 3～4 per week	4 5～6 per week	5 Everytime	portion size S	M	L	0 Never	1 1～2 per month	2 1～2 per week	3 3～4 per week	4 5～6 per week	5 Everytime	portion size S	M	L
Alcohol	50	can of beer 1 cans(500mℓ)	50	0	1	2	3	4	5				0	1	2	3	4	5				0	1	2	3	4	5			
	51	Sake (180ml)	51	0	1	2	3	4	5				0	1	2	3	4	5				0	1	2	3	4	5			
	52	wine 1 glass	52	0	1	2	3	4	5				0	1	2	3	4	5				0	1	2	3	4	5			
	53	Distilled spirits 1/4 glass Shochu hot water allocation / Canned sour 1 glass / whiskey single 1 cup / plum wine 1 glass / 1 glass = 200mℓ	53	0	1	2	3	4	5				0	1	2	3	4	5				0	1	2	3	4	5			
Tea group	54	green tea: sencha·hoji-cha·ban-cha·oolong tea· black tea, 1glass	54	0	1	2	3	4	5				0	1	2	3	4	5				0	1	2	3	4	5			
	55	coffee 1 glass / Canned coffee(sugar-free) 1 cans iced coffee(sugar-free) 1 glass	55	0	1	2	3	4	5				0	1	2	3	4	5				0	1	2	3	4	5			
Confectionaries and soft drinks	56	sport drink 1 glass / carbonated drink 1 glass / soft drinks containing fruit / a lactic acid drink 1 glass / coffee containing sugar 1 cans	56	0	1	2	3	4	5				0	1	2	3	4	5				0	1	2	3	4	5			
	57	UZURAMAME 1 portion size / KONBUMAME 1 portion size / red beans 1 table spoon	57	0	1	2	3	4	5				0	1	2	3	4	5				0	1	2	3	4	5			
	58	1 sweet bun(ann bun·cream bun·jam bun·meron bun·chokokorone·curry bun)	58	0	1	2	3	4	5				0	1	2	3	4	5				0	1	2	3	4	5			
	59	1shortcake·chocolate cake pie·tart·donut / Pound Cake thickness of 3 cm / 1 Cream puff	59	0	1	2	3	4	5				0	1	2	3	4	5				0	1	2	3	4	5			
	60	1 MANJYUU YOUKAN: thickness of 1 / CASTELLA thickness of 1 cm / DORAYAKI·MONAKA·DAIHUKU One third	60	0	1	2	3	4	5				0	1	2	3	4	5				0	1	2	3	4	5			
	61	1 pudding 小1個 / 1 jelly·bavarian cream / 1 sherbet	61	0	1	2	3	4	5				0	1	2	3	4	5				0	1	2	3	4	5			
	62	chocolate 3 pieces / chocolate confectionery	62	0	1	2	3	4	5				0	1	2	3	4	5				0	1	2	3	4	5			
	63	2 fried rice crackers / KARINTOU(black·white) / Crisps·popcorn·Corn snack One hand 1 cups	63	0	1	2	3	4	5				0	1	2	3	4	5				0	1	2	3	4	5			
	64	2 ames·candies / 2 caramels / 2 butterscotches	64	0	1	2	3	4	5				0	1	2	3	4	5				0	1	2	3	4	5			
	65	1 cup ice cream / 1 ice bar / 1 soft cream	65	0	1	2	3	4	5				0	1	2	3	4	5				0	1	2	3	4	5			
	66	1 hard baked rice cracker / 2 rice crackes / 2 KAKIMOTIS	66	0	1	2	3	4	5				0	1	2	3	4	5				0	1	2	3	4	5			
	67	2 cookies / 1 sabouret / 3 biscuits	67	0	1	2	3	4	5				0	1	2	3	4	5				0	1	2	3	4	5			
soup	68	Miso soup 1 portion size	68	0	1	2	3	4	5				0	1	2	3	4	5				0	1	2	3	4	5			
	69	Sumasi soup·vegitable soup·Kentin soup 1 portion size	69	0	1	2	3	4	5				0	1	2	3	4	5				0	1	2	3	4	5			
	70	Potage·Instant soup 1 portion size	70	0	1	2	3	4	5				0	1	2	3	4	5				0	1	2	3	4	5			
Dishes made with sugar and soy sauce	71	sugar·honey·jam one teaspoon / stick sugar 1 piece (3g) / Canned coffee containing sugar half size	71	0	1	2	3	4	5				0	1	2	3	4	5				0	1	2	3	4	5			
	72	Cooked food(pumpkin·Potatoes)· KINPIRAGOBOU	72	0	1	2	3	4	5				0	1	2	3	4	5				0	1	2	3	4	5			
	73	Over soy sauce·Ponzu sauce for vegetables boiled or Sasimi one teaspoon	73	0	1	2	3	4	5				0	1	2	3	4	5				0	1	2	3	4	5			
	74	source·ketchup one tablespoon	74	0	1	2	3	4	5				0	1	2	3	4	5				0	1	2	3	4	5			

※ The next food lists are questionnaires for frequency of use of fats and oiles. Please anser again cuisines that you already ansered the questions.

		"Medium" 1 portion size (for example)	Frequency	Breakfast （including the morning snack）									Lunch （including the afternoon snack）									Dinner （including the night snack）								
				0 Never	1 1～2 per month	2 1～2 per week	3 3～4 per week	4 5～6 per week	5 Everytime	portion size S	M	L	0 Never	1 1～2 per month	2 1～2 per week	3 3～4 per week	4 5～6 per week	5 Everytime	portion size S	M	L	0 Never	1 1～2 per month	2 1～2 per week	3 3～4 per week	4 5～6 per week	5 Everytime	portion size S	M	L
Fats and oils	75	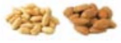 Peanuts･Almond･Cashew nuts One hand 1 cup 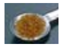 Sesame one tablespoon	75	0	1	2	3	4	5				0	1	2	3	4	5				0	1	2	3	4	5			
	76	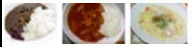 Curry and rice･Hayashi rice･Stew 1 portion size	76	0	1	2	3	4	5				0	1	2	3	4	5				0	1	2	3	4	5			
	77	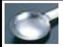 Mayonnaise･dressing one tablespoon 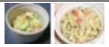 Potato salad･Macaroni salad 1 portion size	77	0	1	2	3	4	5				0	1	2	3	4	5				0	1	2	3	4	5			
	78	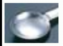 Non-oil dressing one tablespoon	78	0	1	2	3	4	5				0	1	2	3	4	5				0	1	2	3	4	5			
	79	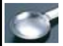 Butter･Margarine one tablespoon 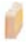 Butter･Margarine one piece 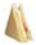 Toast･Sandwich 1 portion size	79	0	1	2	3	4	5				0	1	2	3	4	5				0	1	2	3	4	5			
	80	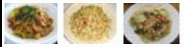 Fried noodles･Fried Rice･Stir-fried rice noodles	80	0	1	2	3	4	5				0	1	2	3	4	5				0	1	2	3	4	5			
	81	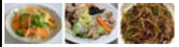 Fried vegetables 1portion size Chinese-style fried 1portion size	81	0	1	2	3	4	5				0	1	2	3	4	5				0	1	2	3	4	5			
	82	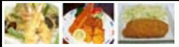 Tenpura･Fried food 1portion size 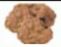 Fried chikin 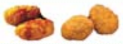 Fried frozen food 2 pieces	82	0	1	2	3	4	5				0	1	2	3	4	5				0	1	2	3	4	5			
