# Supplementary material for: Effect of School-Based Home-Collaborative Lifestyle Education on Reducing Subjective Psychosomatic Symptoms in Adolescents: A Cluster Randomised Controlled Trial
Source: PLoS One. 2016 Oct 25;11(10):e0165285. doi: 10.1371/journal.pone.0165285 (PMC5079616; doi:10.1371/journal.pone.0165285)
Supplement: S1 Table — SPS, subjective psychosomatic symptoms; ITT/MI, Analysis by intention-to-treat principles using multiple imputation (number of imputations = 200); SE, standard error; Model 1, crude mixed model; Model 2, mixed model adjusted for baseline; Model 3, mixed model adjusted for baseline, sex, age, and BMI. (PDF) [file pone.0165285.s005.pdf]

S1 Table. Mean change of the SPS score from baseline at 6 months (intervention effect on primary outcome) by multiple imputation.

**Total**	**ITT/MI (n = 1,509)**				
	**Difference**	**SE**	**95% CI**		**P-value**
**Model 1**	−1.02	0.48	−2.05	−0.003	0.049
**Model 2**	−0.78	0.47	−1.78	0.22	0.118
**Model 3**	−0.79	0.49	−1.84	0.28	0.131

SPS, subjective psychosomatic symptoms; ITT/MI, Analysis by intention-to-treat principles using multiple imputation (number of imputations = 200); SE, standard error; Model 1, crude mixed model; Model 2, mixed model adjusted for baseline; Model 3, mixed model adjusted for baseline, sex, age, and BMI.
