# Supplementary material for: Effect of School-Based Home-Collaborative Lifestyle Education on Reducing Subjective Psychosomatic Symptoms in Adolescents: A Cluster Randomised Controlled Trial
Source: PLoS One. 2016 Oct 25;11(10):e0165285. doi: 10.1371/journal.pone.0165285 (PMC5079616; doi:10.1371/journal.pone.0165285)
Supplement: S2 Table — SPS, subjective psychosomatic symptoms; ITT/LOCF, Analysis by intention-to-treat principles performing imputation of missing data using the last observation carried forward method; SE, standard error; Model 1, crude mixed model; Model 2, mixed model adjusted for baseline; Model 3, mixed model adjusted for baseline, sex, age, and BMI. (PDF) [file pone.0165285.s006.pdf]

S2 Table. Secondary analysis: Mean change of the SPS-D score from baseline at 6 months for ITT/LOCF (intervention effect on primary outcome).

SPS-D score	ITT/LOCF (n = 1,509)				
	Difference	*SE*	95% CI		P-value
Model 1	−0.53	0.18	−0.90	−0.16	0.008
Model 2	−0.50	0.15	−0.82	−0.18	0.004
Model 3	−0.49	0.17	−0.84	−0.14	0.009

SPS, subjective psychosomatic symptoms; ITT/LOCF, Analysis by intention-to-treat principles performing imputation of missing data using the last observation carried forward method; SE, standard error; Model 1, crude mixed model; Model 2, mixed model adjusted for baseline; Model 3, mixed model adjusted for baseline, sex, age, and BMI.
