# Supplementary material for: Effect of School-Based Home-Collaborative Lifestyle Education on Reducing Subjective Psychosomatic Symptoms in Adolescents: A Cluster Randomised Controlled Trial
Source: PLoS One. 2016 Oct 25;11(10):e0165285. doi: 10.1371/journal.pone.0165285 (PMC5079616; doi:10.1371/journal.pone.0165285)
Supplement: S3 Table — SPS, subjective psychosomatic symptoms; PPS, Analysis by per protocol set with the complete data set; SE, standard error; Model 1, crude mixed model; Model 2, mixed model adjusted for baseline; Model 3, mixed model adjusted for baseline, sex, age, and BMI. (PDF) [file pone.0165285.s007.pdf]

S3 Table. Sensitivity analysis: Mean change of the SPS and SPS-D scores from baseline at 6 months (intervention effect on primary outcome).

**Total**	**PPS (n = 1,420) for SPS**					**PPS (n = 1,420) for SPS-D**				
	**Difference**	**SE**	**95% CI**		**P value**		**SE**	**95% CI**		**P-value**
**Model 1**	−0.99	0.42	−1.89	−0.10	0.032	−0.57	0.19	−0.98	−0.16	0.009
**Model 2**	−0.74	0.45	−1.68	0.20	0.115	−0.54	0.16	−0.88	−0.19	0.005
**Model 3**	−0.77	0.47	−1.76	0.22	0.122	−0.53	0.18	−0.91	−0.16	0.008

SPS, subjective psychosomatic symptoms; PPS, Analysis by per protocol set with the complete data set; SE, standard error; Model 1, crude mixed model; Model 2, mixed model adjusted for baseline; Model 3, mixed model adjusted for baseline, sex, age, and BMI.
