# Supplementary material for: Effect of School-Based Home-Collaborative Lifestyle Education on Reducing Subjective Psychosomatic Symptoms in Adolescents: A Cluster Randomised Controlled Trial
Source: PLoS One. 2016 Oct 25;11(10):e0165285. doi: 10.1371/journal.pone.0165285 (PMC5079616; doi:10.1371/journal.pone.0165285)
Supplement: S4 Table — PPS, Analysis by per protocol set with the complete data set; OR (odds ratio) < 1 indicates favourable for resolving lifestyle factors; Model 2, mixed model adjusted for baseline; Model 3, mixed model adjusted for baseline, sex, age, and BMI; 1) Staple food: rice, bread, etc.; 2) Main dishes: fish, soy, eggs, meat, etc. (PDF) [file pone.0165285.s008.pdf]

S4 Table. Sensitivity analysis: Effects of lifestyle change at 6 months on resolving lifestyle factors (intervention effects on secondary outcomes; baseline-adjusted odds ratio for PPS [Models 2 & 3]).

Lifestyle factors	PPS (n = 1,420)							
	Model 2				Model 3			
	OR	95% CI Lower	95% CI Upper	P-value	OR	95% CI Lower	95% CI Upper	P-value
**Enjoying school life (very much)**	0.49	0.28	0.86	0.013	0.47	0.28	0.81	0.006
**Taking exercise and stretching**	1.01	0.73	1.39	0.936	1.01	0.74	1.38	0.970
**Fast sleep at 12 AM (midnight)**	0.94	0.61	1.46	0.799	0.94	0.66	1.33	0.708
**More than 6 hours sleep**	0.88	0.68	1.13	0.293	0.85	0.66	1.08	0.187
**Health condition (very good)**	0.87	0.64	1.17	0.359	0.82	0.60	1.13	0.221
**Staple food[1)] consumed per breakfast**	0.66	0.47	0.91	0.014	0.60	0.43	0.86	0.005
**Main dishes[2)] consumed per breakfast**	0.73	0.53	0.99	0.043	0.70	0.53	0.93	0.015
**Vegetables consumed per breakfast**	0.66	0.45	0.96	0.031	0.64	0.46	0.89	0.008
**Main dishes consumed per lunch**	0.95	0.59	1.52	0.835	0.96	0.61	1.53	0.872
**Vegetables consumed per lunch**	1.19	0.85	1.65	0.306	1.19	0.86	1.64	0.298
**Dairy products consumed per day**	0.80	0.55	1.17	0.261	0.81	0.60	1.11	0.192
**Not consumed fatty foods**	0.94	0.64	1.36	0.734	0.91	0.64	1.32	0.628
**Not consumed snacks after 10 PM**	1.12	0.82	1.52	0.484	1.08	0.79	1.49	0.621

PPS, Analysis by per protocol set with the complete data set; OR (odds ratio) < 1 indicates favourable for resolving lifestyle factors; Model 2, mixed model adjusted for baseline; Model 3, mixed model adjusted for baseline, sex, age, and BMI; 1) Staple food: rice, bread, etc.; 2) Main dishes: fish, soy, eggs, meat, etc.
